# Nighttime Organic Nitrates Drive New Particle Formation and Aerosol Growth in Urban Beijing

**DOI:** 10.1021/acs.est.6c01200

**Published:** 2026-07-07

**Authors:** Junfeng Wang, Eleonora Aruffo, Jianhuai Ye, Xinlei Ge, Scot T. Martin, Yele Sun, Weiqi Xu, Yuepeng Pan, Alessandra Mascitelli, Piero Chiacchiaretta, Jie Zhang, Jingyi Li, Yiming Qin, Haiwei Li, Ke Li, Ning Zhang, Ming Wang, Chunxiang Ye, Jianbo Zhang, Wei Tao, Tzung-May Fu, Hong Liao, Pingqing Fu, Qi Zhang, Mindong Chen, Jingkun Jiang, Hugh Coe, David Topping, James Lee, Piero Di Carlo, Daniel J. Jacob

**Affiliations:** † Jiangsu Key Laboratory of Intelligent Atmospheric Environment Monitoring and Carbon–Pollution Co-control, Collaborative Innovation Center of Atmospheric Environment and Equipment Technology (CIC-AEET), School of Environmental Science and Engineering, 71127 Nanjing University of Information Science and Technology, Nanjing 210044, China; ‡ John A. Paulson School of Engineering and Applied Sciences, 1812 Harvard University, Cambridge, Massachusetts 02138, United States; § Department of Science, Center for Advanced Studies and Technology (CAST), University “G. d’Annunzio” of Chieti-Pescara, 66100 Chieti, Italy; ∥ Guangdong Provincial Observation and Research Station for Coastal Atmosphere and Climate of the Greater Bay Area (GORSCAC), School of Environmental Science & Engineering, 255310 Southern University of Science and Technology, Shenzhen 518055, China; ⊥ School of Energy and Environment, Southeast University, Nanjing 211189, China; # State Key Laboratory of Atmospheric Boundary Layer Physics and Atmospheric Chemistry, Institute of Atmospheric Physics, Chinese Academy of Sciences, Beijing 100029, China; 7 Department of Advanced Technologies in Medicine & Dentistry, Center for Advanced Studies and Technology (CAST), University “G. d’Annunzio” of Chieti-Pescara, 66100 Chieti, Italy; 8 Atmospheric Sciences Research Center, University at Albany, State University of New York, Albany, New York 12203, United States; 9 School of Energy and Environment, City University of Hong Kong, Kowloon, Hong Kong 999077, China; 10 State Key Joint Laboratory of Environmental Simulation and Pollution Control, College of Environmental Sciences and Engineering, 12465 Peking University, Beijing 100080, China; 11 Institute of Surface-Earth System Science, 12605 Tianjin University, Tianjin 300072, China; 12 Department of Environmental Toxicology, University of California, Davis, Davis, California 95616, United States; 13 School of Environment, Tsinghua University, Beijing 100084, China; 14 School of Earth and Environmental Science, 5292 University of Manchester, Manchester M13 9PL, United Kingdom; 15 Department of Chemistry, University of York, York YO10 5DD, United Kingdom

**Keywords:** secondary organic aerosol, organic nitrates, nighttime chemistry, new particle formation, vertical transport

## Abstract

Secondary organic aerosol (SOA) represents a major component of urban air pollution. This study presents observational evidence from summer 2017 in urban Beijing, supported by model simulations and a case study from summer 2023, demonstrating the crucial role of nighttime organic nitrates (ONs) production in subsequent daytime SOA formation. Our measurements revealed that total reactive nitrogen compound (NO_*z*_) concentrations exceeded 40 ppb at night, resulting from nitrate radical (NO_3_)-initiated oxidation of volatile organic compounds (VOCs) in the surface layer and through aloft production followed by downward transport. While these NO_*z*_ existed primarily in the gas phase during nighttime, they underwent atmospheric aging processes the following day, significantly contributing to SOA growth and potentially new particle formation. Model simulations identified reactive terpenoids as the dominant VOC precursors for nighttime ON formation. These findings underscore the need for an improved understanding of nocturnal ONs production mechanisms given their substantial impact on daytime SOA production.

## Graphical abstract

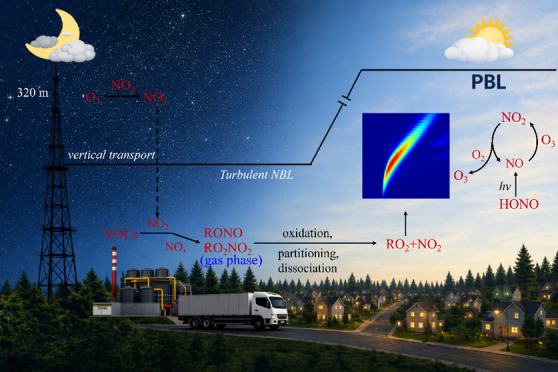

## Introduction

Organic nitrates (ONs) are key atmospheric constituents that significantly influence air quality, climate, and human health.^1–7^ These compounds mainly include alkyl nitrates (ANs, RONO_2_) and peroxy nitrates (PNs, RO_2_NO_2_), which are primarily produced by reactions of organic radicals with nitrogen oxides (NO_*x*_ ≡ NO + NO_2_) during daytime.^8^ At night, ONs production can also occur via reactions of volatile organic compounds (VOCs) with nitrate radicals (NO_3_),^3^ substantially contributing to nocturnal atmospheric oxidation processes.

ONs are ubiquitous in the atmosphere. Field studies across forty-seven Chinese cities show that the most abundant short-chain ONs (C1–C4) in ambient air include 2-butyl nitrate and 2-propyl nitrate, highlighting the prevalence of alkyl nitrates in densely populated areas.^9^ Peroxyacetyl nitrate (PAN), another important gaseous organic nitrate, can accumulate to concentrations of tens of ppb during summertime in urban Beijing, making it a critical reactive nitrogen reservoir species and a key contributor to regional photochemical smog.^10^ In regard to particulate organic nitrates (pONs), literature estimated that pONs can account for 10–100% of total nitrogen-containing compounds across different regions globally.^11^ In China, pONs represent a nontrivial component of ambient aerosol. Snow samples collected from four different Chinese megacities revealed that the nitrogen-containing organic compounds were mainly present in oxidized form as ONs.^12^ Studies showed that pONs constituted 15–28% of organic aerosol in the Beijing–Tianjin–Hebei region.^13^ Even in the relatively cleaner Pearl River Delta region, where total organic aerosol concentrations are significantly lower than in other parts of China, the proportional contribution of pONs remains substantial. For example, Yu et al. reported that pONs can account for up to 25% of total organic aerosol in Shenzhen (China), significantly influencing the physicochemical properties of atmospheric particulate matter.^14^

ONs are key drivers in urban atmospheric chemistry. The formation of ONs terminates the chain reactions of RO_*x*_ (≡OH + HO_2_ + RO_2_) and NO_*x*_. They thereby modulate the concentrations of OH and O_3_ in the daytime and NO_3_ radical at night, further regulating atmospheric oxidative capacity.^1^ In particle phase, ONs provide an important pathway for nitrogen participating in secondary organic aerosol (SOA),^15^ contributing to particle mass and influencing their physicochemical properties such as light absorption, hygroscopicity, and heterogeneous reactivity, which in turn affect air quality and aerosol–cloud interactions.

ONs are also important components of atmospheric brown carbon. Chamber experiments found that organonitrates as possible chromophores in BrC from pyrrole, furan, and thiophene precursors in nighttime.^16^ Laboratory simulations revealed that anthropogenic VOCs such as toluene can form substantial amounts of pONs through photochemical oxidation in the presence of NO_*x*_. These pONs are major contributors to the light-absorbing properties of aromatic oxidation products.^6,17,18^ Furthermore, pONs can enhance the light absorption of secondary products from biogenic sources. Studies on the nighttime NO_3_ oxidation of monoterpenes and other biogenic VOCs have identified organic nitrate monomers and dimers as significant light-absorbing species.^19^

In addition to their environmental and climatic roles, the toxicological properties of ONs are a major focus in environmental health research. Reactive nitrogen species in pONs can trigger inflammatory cellular responses.^20^ Inhalation of nitrated organic compounds may induce immune reactions and promote allergic diseases.^7^ Organic nitrate esters may undergo hydrolysis in human body to form nitric acid, which can impair lung function.^21^

Collectively, these findings emphasize that ONs, in both gaseous and particulate phases, constitute a substantial fraction of reactive nitrogen and organic aerosol with significant environmental, climatic, and health impacts, and represent an important but under-constrained component of atmospheric pollution. Here, we present detailed observations of ONs in summer Beijing to elucidate their formation pathways and implications in linking atmospheric NO_*x*_ cycling, oxidant budget, and particle production in complex urban environments. The results show that nighttime chemistry is a large source of gas-phase ONs. These ONs are then oxidized the following day, leading to SOA production. We propose that this nighttime chemistry is driven both by NO_3_ at the surface or transported from aloft. Such processes highlight the dynamic interplay between nighttime NO_3_ oxidation and daytime photochemistry, yet they are currently absent from most atmospheric chemistry models, leading to underestimation of ONs contributions to oxidant and aerosol budgets in polluted regions.

## Materials and Methods

### Field Campaigns

The data presented here are from the Atmospheric Pollution and Human Health in a Chinese Megacity (APHH-Beijing).^46^ The summer campaign was carried out at the Tower Division of the Institute of Atmospheric Physics, Chinese Academy of Science (39° 58′ N, 116° 22′ E) in Beijing, China, from June 4 to 20, 2017, and June 2023. All data presented in this paper were hourly averaged (Beijing time, UTC + 8).

NO_*y*_, NO, NO_2_, and O_3_ concentrations were measured by the Thermo Fisher Scientific instruments (42C, 42i, and 49i), respectively. Based on previous studies in summer Beijing,^22^ HNO_3_ contribution to NOy can be considered negligible so that we expect NO_*y*_ to represent the sum of nitrogen oxides less HNO_3_. NO_2_ was also sampled by a Teledyne CAPS system. OH, HO_2_, and RO_2_ radical concentrations were measured by a fluorescence assay by gas expansion (FAGE). A broadband cavity-enhanced absorption spectroscopy (BBCEAS) system was employed to measure HONO, NO_3_, and N_2_O_5_ concentrations. Individual VOCs and OVOCs were collected by a PTR-MS. PAN was observed by the GC-ECD instrument. NO_*z*_ (NO_*y*_ – NO_*x*_) was derived from independent measurements of NO_*y*_, NO, and NO_2_ by Thermo Scientific model 42i-Y NO_*y*_, model 42i-TL trace level NO_*x*_ analyzer, and Teledyne T500U CAPS analyzer, respectively.

An Aerodyne high-resolution aerosol mass spectrometer (HR-AMS) was deployed to measure the chemical composition of nonrefractory submicron particulate matter (NR-PM_1_). pONs were estimated by the different ratios of NO^+^/NO_2_^+^ between particulate organic nitrates and ammonium nitrate.^13,15^ In parallel, aerosol size distribution was obtained by a standard TSI scanning mobility particle sizer (SMPS) and a long-differential mobility analyzer (DMA) SMPS. Meteorological parameters were also collected simultaneously at the site.

In the summer of 2023, a new field campaign was carried out employing a thermal dissociation laser-induced fluorescence (TD-LIF) instrument to measure the NO_2_ and alkyl nitrates (RONO_2_) and the peroxy nitrates (RO_2_NO_2_) in both gas and particle phases, by a charcoal denuder. The campaign took place at the same site as the APHH campaign in Beijing, China from June 1 to 30, 2023. Details about the TD-LIF instrument can be found elsewhere.^47^ A Vocus PTR-MS (TOFWERK, Switzerland) was conducted parallelly to collect individual VOC species, details of the configuration of the Vocus PTR-MS can be found a previous work.^48^

### F0AM–WAM Simulations

Local air chemistry was simulated using the framework for 0D atmospheric modeling (F0AM), a 0D box model based on MATLAB^49^ to simulate gas phase. The simulations were constrained using NO, NO_2_, O_3_, individual VOC species (Table S2), CO, NO_3_, N_2_O_5_, and HONO measurement data sets during the APHH-Beijing summer field campaign. The meteorological parameters of pressure, temperature, and relative humidity were also included as inputs of the model. Photochemistry was simulated by using the solar zenith angle (SZA) trigonometric function in the F0AM model. We included a residual 10 ppb HNO_3_ in the F0AM simulations to account for nighttime nitric acid levels influencing organic nitrate chemistry. To simulate the ONs in both stage I and II, as shown in Figure 3 and Figures S7 and S10, we used simply the basic F0AM model.

The F0AM–WAM simulations have been constrained by using some of the species given as output of the aforesaid simulations by the F0AM, which simulated only the gas phase. In detail, we used the same input employed in simple F0AM simulations adding to them the modeled 2-oxopropyl nitrate, PAN, and methylglyoxal. We set 100 seeds per cm^3^ with a radius of 25 × 10^–7^ cm as initial values to initiate the particle simulation. The WAM is a custom dynamic gas-particle partitioning module that explicitly resolves condensation and evaporation kinetics. Gas-to-particle transfer is governed by diffusion-limited mass transport and a mass accommodation coefficient of 0.1, while compound-specific saturation concentrations determine reversible uptake. We used the WAM module only to independently confirm the daytime production and subsequent growth of particles, as shown in Figure S9.

## Results and Discussion

### Field Observations

Measurements were made at a rooftop lab 8 m above street level of the Institute of Atmospheric Physics, Chinese Academy of Sciences, in Beijing, in June 2017. The temporal variations of the concentrations of key species are presented in Figure 1. Concentrations of NO, NO_2_, and total nitrogen oxides (NO_*y*_) were measured. A useful quantity for our analysis is NO_*z*_ (≡NO_*y*_ – NO_*x*_), representing the sum of gas- and particle-phase NO_*x*_ oxidation products. Previous campaigns in summer Beijing^22^ have shown that when NO_2_ averaged around 32 ppb, HNO_3_ remained below 1 ppb. Since the conditions during the Beijing summer 2017 campaign were similar to those of previous campaigns, we considered the contribution of HNO_3_ to NO_*y*_ negligible. Indeed, during our June 2023 campaign at the same site in similar conditions, HNO_3_ was observed with a negligible concentration close to zero ppb (Figure S1).

**Figure 1:**
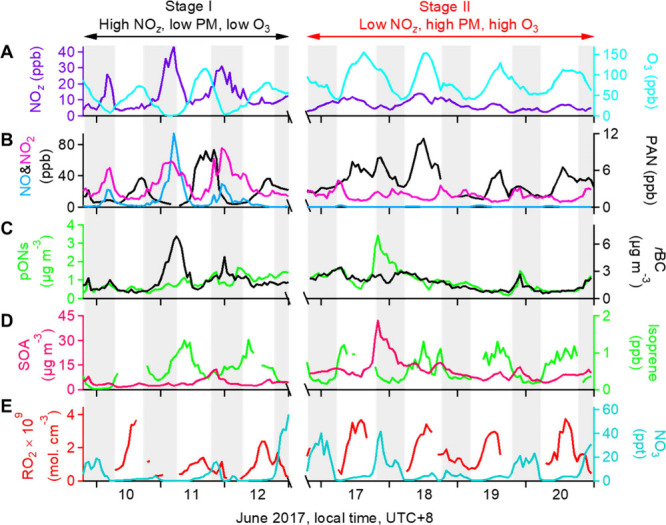
Time series of selected variables during the atmospheric pollution and human health (APHH) summer campaign in Beijing: (A) NO_*z*_ (sum of NO_*x*_ oxidation products) and O_3_; (B) NO, NO_2_, and peroxyacetyl nitrate (PAN); (C) particulate organic nitrates (pONs) and refractory black carbon (*r*BC) in PM_1_ measured by an Aerodyne soot-particle aerosol mass spectrometer; (D) SOA in PM_1_ and isoprene; and (E) total RO_2_ radicals and NO_3_. Nighttime (19:00–6:00) is colored in gray.

Two stages during the campaign were selected based on back-trajectory analyses.^23^ June 9 to 12, denoted as stage I, sampled air from eastern NCP (North China Plain) (Figure S2). Mean concentrations of O_3_ and PM_1_ (PM less than 1 μm diameter) during that stage were relatively low at 44 ± 30 ppb and 15 ± 7 μg m^–3^, respectively. Stage II, covering the period from June 16 to 20, sampled air from western NCP (Figure S2). This time period was characterized by mean concentrations of O_3_ and PM_1_ of 89 ± 31 ppb and 30 ± 13 μg m^–3^, respectively, roughly double the values of stage I, indicating a shift in regional air quality conditions. Similar results have been observed in another campaign at the same site in June 2023 (Figure S1).

### Different Nighttime Nitrate Formation Pathways in the Two Stages

NO_*z*_ had different behavior in stages I and II (Figure 1A). In stage I, NO_*z*_ and O_3_ exhibited a strong anticorrelation at night (*r* = −0.73) (Figure S3A). A maximum NO_*z*_ concentration of 43 ppb and a minimum O_3_ concentration of less than 1 ppb were observed. It is important to note that, although high concentrations of NO were observed in nighttime stage I, the presence of NO_3_ was still detected (Figure 1). By comparison, NO_*z*_ in stage II correlated weakly with O_3_ (*r* = 0.33 at night) (Figure S3B). The maximum nighttime concentration of NO_*z*_ in stage II was <10 ppb, while O_3_ maintained greater than 40 ppb. Interestingly, peroxyacetyl nitrate (PAN), pONs, the nitrogen-to-carbon ratio (N/C) of total organic aerosol, and SOA in PM_1_, all had higher nighttime values in stage II than in stage I (Figures 1C and D and 2; Materials and Methods and Table S1). In addition, higher concentrations of radical oxidants (daytime RO_2_ and nighttime NO_3_) were observed in stage II (Figure 1E and Figure S4). These findings suggest that NO_*z*_ contributed more significantly to particle-phase formation in stage II at night. The lower RO_2_ levels observed in stage I under high-NO_*x*_ conditions suggest that RO_2_ is predominantly consumed through reactions with NO_*x*_, promoting the formation of organic nitrates and consequently yielding less SOA (Figure 1). In contrast, the higher RO_2_ concentrations and low NO_*x*_ levels measured in stage II indicate that RO_2_ is more likely to react with HO_2_ or undergo accretion reactions, pathways that are substantially more efficient at producing SOA (Figure 1).

**Figure 2:**
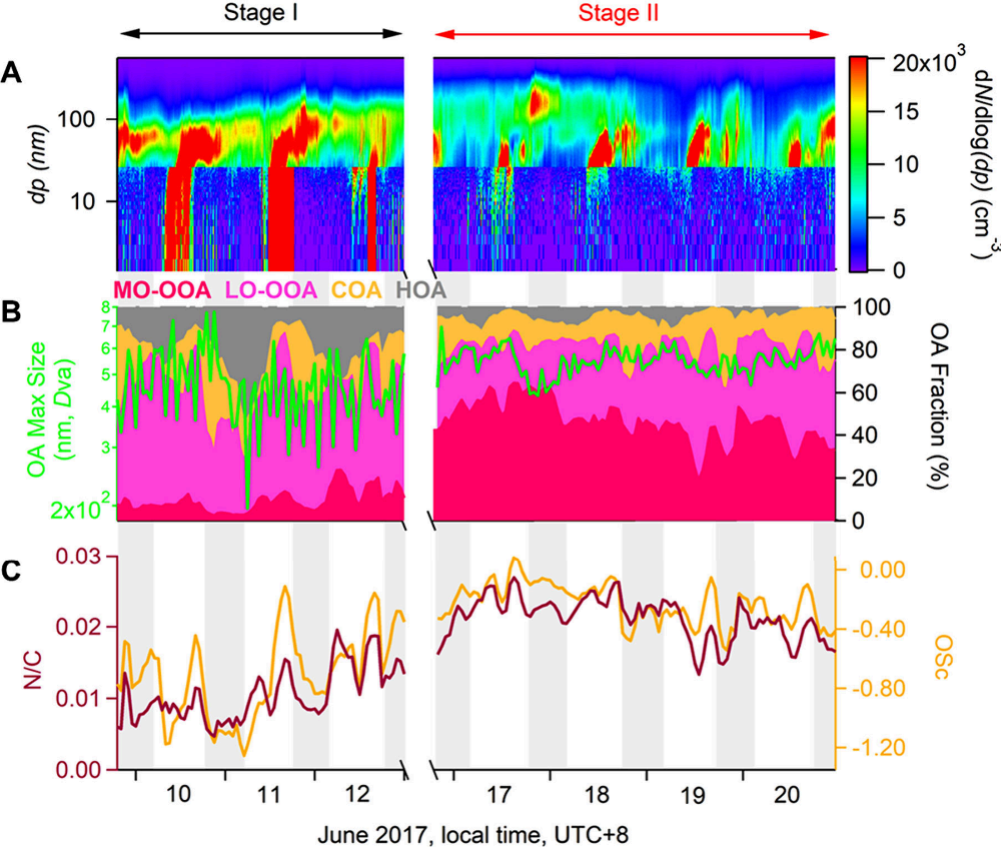
New particle formation (NPF) events during stages I and II of the APHH-Beijing summer campaign: (A) scanning mobility particle sizer (SMPS) measured size distributions with NPF events identified as surges in the concentrations of <10 nm particles; (B) Aerodyne AMS measured peak diameters of OA size distributions and fractions of four OA factors (more oxidized oxygenated MO-OOA), less oxidized oxygenated OA (LO-OOA), cooking OA (COA), and hydrocarbon-like OA (HOA)) identified by positive matrix factorization (PMF); and (C) Aerodyne HR-AMS measured nitrogen to carbon ratios (N/C) in PM_1_ and OA oxidation state (OSc = 2 × O/C – H/C). Gray boxes represent the nighttime.

The evolution of NO_*z*_ exhibited distinct characteristics between the two stages, reflecting different chemical regimes and formation pathways (Figure S5). In stage I, NO_*z*_ concentration showed strong nocturnal production closely linked to local NO_*x*_ emissions (*r* = 0.94, Figure S3C), indicating rapid nighttime oxidation of freshly emitted NO_*x*_ under conditions that favored gas-phase products; this was evidenced by minimal contributions of PAN and pONs to total NO_*z*_ (<2%), suggesting the formation of less functionalized compounds that remained predominantly in the gas phase. Further aerosol composition analysis in stage I supported this interpretation, with PM_1_ showing relatively low oxidation state (OSc), minimal contribution from more-oxidized oxygenated organic aerosol (MO-OOA), and elevated levels of refractory black carbon (*r*BC), while total secondary organic aerosol (SOA as LO-OOA + MO-OOA) reached only 12 μg m^–3^ (lower than the average value during the campaign, Figure 1). In marked contrast, stage II exhibited fundamentally different NO_*z*_ chemistry, where the loss of correlation with NO_*x*_ implied regional production and transport, while PAN became the dominant component (slope ≈ 1 against NO_*z*_ at night) and pONs contributed a significantly greater fraction (10%) of total NO_*z*_, signatures of more aged and processed air masses. This shift toward more functionalized compounds was further reflected in the aerosol phase through enhanced partitioning to particles during daytime, consistent with the observed increases in both MO-OOA contribution and overall SOA loading (peaking at 42 μg m^–3^), along with higher overall oxidation state, demonstrating the progression from locally dominated, night-focused chemistry in stage I to regionally influenced, daytime-driven oxidation processes in stage II.

Nighttime ONs are expected to be produced at the surface level via O_3_ reacting with alkenes in the presence of NO_*x*_. Additionally, ONs can be produced at night via reactions between NO_3_ and VOCs, directly generating nitrated alkylperoxy radicals.^24–26^ NO_3_ is produced by the reaction between O_3_ and NO_2_, which itself is formed by oxidation of NO by O_3_. In stage I at night, O_3_ in surface air was titrated by fresh NO emissions (Figure 1 and Figure S5D). The formation of NO_3_ was limited, consistent with the observed low NO_3_ concentration (Figure 1E). However, significant NO_3_ production may still occur in the residual layer, where elevated O_3_ and relatively high NO_2_ concentrations were present,^27,28^ facilitating ON formation. The surface layer (occurring intermittently during nighttime and more prominently during the transitional periods immediately preceding and following sunrise) was sustained by mechanical turbulence extending from 50 to 500 m above ground level, as evidenced by vertical wind speed profiles (Figure S6A). Under these conditions, model simulations demonstrate that NO_3_ generated in the residual layer could be transported downward to the surface layer through either mechanical turbulence or concentration gradient-driven diffusion (Text S1 and Figure S6B). Similarly, residual layer-produced ONs may also undergo downward mixing into the surface layer. The confinement of high NO and suppressed O_3_ within the shallow, stably stratified nocturnal surface layer-where fresh emissions rapidly titrate O_3_,contrasts sharply with the chemically active residual layer aloft. This decoupled layer preserves late-afternoon O_3_ and NO_2_, sustaining a reservoir of precursors for nocturnal NO_3_ production via O_3_ + NO_2_. Such vertical segregation is well documented in urban airsheds, including the North China Plain.^29,30^ Nocturnal low-level jets and intermittent mechanical mixing can erode the stable boundary layer,^31^ episodically transporting O_3_, NO_2_, and potentially NO_3_ radicals downward. These dynamics support the assumption of elevated oxidant levels aloft, with the residual layer functioning as a persistent nocturnal chemical reservoir capable of intermittently coupling with the surface.

#### ONs Formation in Stage I

The co-occurrence of high gas-phase NO_*z*_ concentrations and low particle number concentrations during the night in stage I (Figure 1) suggests these conditions may have triggered new particle formation (NPF) events the following day, where less oxidized oxygenated organic aerosol (OA) was the dominant OA species (Figure 2A and B).^32^ The RONO_2_ produced at night could undergo photochemical functionalization in the daytime, decreasing their vapor pressure and leading to NPF and further to particle growth.^33^ The daytime increase in the OA N/C ratio (Figure 2C and Figure S4) provides evidence for the contribution of ONs to the growth of particles. This rapid oxidation of NO_*x*_ to NO_*z*_ was also reported for nighttime Houston urban plumes,^34^ and NO_3_ chemistry was identified as a driver for the production of ONs and SOA.^35^ Although urban NPF is typically dominated by sulfuric acid–base chemistry, the low sulfate concentrations observed during stage I in 2017 (not shown) suggest that the canonical acid-ammonia nucleation pathway was likely suppressed. Under these conditions, the abundant ONs present in our measurements could efficiently condense onto and grow freshly nucleated clusters, thereby acting as the dominant driver of early particle growth into the detectable size range. Recent studies have shown that ONs may also participate directly in the initial nucleation step. Field observations in the Amazon^33^ and CLOUD chamber experiments^36^ demonstrate that isoprene-derived ONs can nucleate under cold upper tropospheric conditions, particularly in synergy with trace inorganic acids. Whether such ONs-mediated nucleation pathways operate in the warmer, more chemically complex, and polluted urban boundary layer remains uncertain. Nonetheless, the uniquely low-sulfate regime of stage I highlights the need to reassess the potential contribution of ONs to the earliest stages of NPF in nontraditional urban environments.

A zero-dimension box model (F0AM), based on the master chemistry mechanism (MCM) (Materials and Methods), was used to assess the dominant processes governing the formation of nighttime gas-phase ONs in stage I. The simulations were constrained by the summer data set of O_3_, NO, NO_2_, NO_3_, OH, HONO, and independent VOC species (Table S2). The F0AM model does not reproduce the ONs peak at 6:00 AM based on the measured NO_3_ (Figure 3A and B). We therefore hypothesized that additional NO_3_ or ONs were transported from aloft, based on flux observations (Figure S6). Adding these sources in the simulation reproduced the nighttime peak of the RONO_2_ (Figure 3C). Furthermore, the simulations showed that biogenic alkenes could play a crucial role in explaining the nighttime production of ONs compared to other VOCs. With the observed VOC input, the model well captured NO_*z*_ formation and evolution at night (Figure S7B and C), with dominant ONs species such as C_3_H_5_O–ONO_2_ and C_5_H_7_O–ONO_2_ produced from isoprene oxidation and C_9_H_15_O–ONO_2_ from monoterpene oxidation (Table S2 and Figure S7A). The main mechanism for isoprene and α-pinene derived ONs production at night is illustrated in Figure S8.

**Figure 3:**
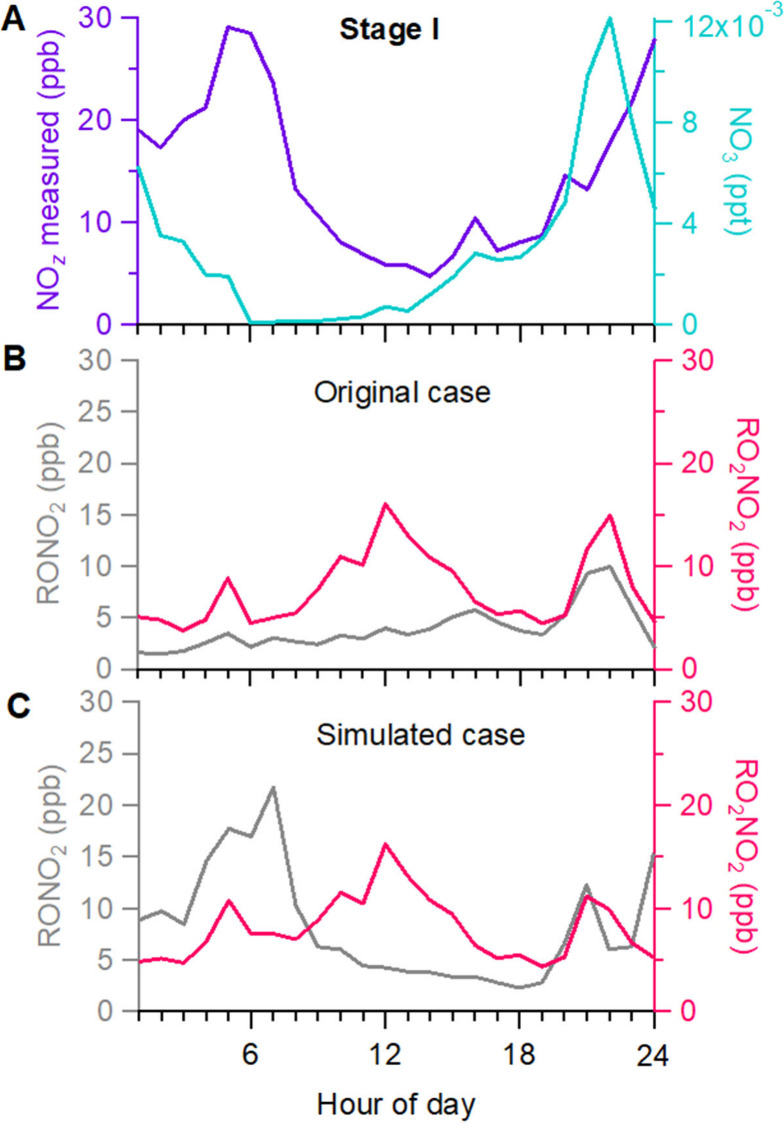
F0AM-MCM model simulations for stage I: (A) measured NO_*z*_ and the measured and transported NO_3_; (B) RONO_2_, and RO_2_NO_2_ simulated by using the measured concentration of NO_3_; and (C) similar to panel (B) but with the hypothesized transported trend of NO_3_.

The model results support that RONO_2_ at night was produced mainly by the oxidation of reactive terpenoids by NO_3_ (Figure 3C), consistent with previous observations.^37^ Hu et al.^38^ reported that isoprene was the dominant species for NO_3_ reactivity during the summer in urban Beijing (up to 80%). Yang et al.^39^ indicated that isoprene played a key role in the nighttime NO_3_ chemistry in a suburban site in Beijing. The nighttime production of gas phase RONO_2_ in the presence of high NO concentration with little pre-existing PM (i.e., similar conditions found in stage I in Beijing) has been previously suggested based on smog chamber experiments.^32^

To further verify the daytime production and growth of particles, we used the F0AM model integrated with the Washington Aerosol Module (WAM).^40^ Figure S9 shows that the observed SOA formation and growth in stage I can be effectively modeled and reproduced during the daytime (peaked at midday). The NO_*z*_ undergoes functionalization when photochemistry is activated, thereby enhancing the partitioning of oxidation products into the particle phase, and promoting the formation and growth of daytime SOA (Figure 2A). The findings and analyses discussed above are further substantiated by the field campaign conducted in June 2023 at the same site in Beijing (Figure 4A). The meteorological situation on June 11, 2023 is similar to stage I in June 2017 (Figure S1). In stage I, the temperature is 23.8 ± 4.1 °C, closely matching the conditions on June 11, 2023 (23.6 ± 3.1 °C). The RH differs with values of 26.9 ± 8.8% in stage I compared with 50.5 ± 12.2% on June 11, 2023. In both cases, the wind direction is consistent, with air masses arriving from the southeast. In the summer 2023, ONs were directly sampled by using a thermal dissociation laser-induced fluorescence (TD-LIF) instrument. This instrument provided ONs speciation by distinguishing between RONO_2_ and RO_2_NO_2_. It also measured gas-to-particle partitioning by separately measuring gas and particle-phase products.^32^ Figure 4B shows that the directly measured gas phase RONO_2_ (gRONO_2_) had a peak between 3:00 and 6:00 AM. The particle phase (pRONO_2_) had a peak at around 10:00 AM, which was synchronous with an NPF event as shown in Figure 4A. Literature also presented comprehensive in situ aircraft measurements demonstrating that highly oxidized isoprene derivatives, especially specific organonitrate compounds with extremely low volatility, can initiate NPF in the upper troposphere above the Amazon rainforest.^33^

**Figure 4:**
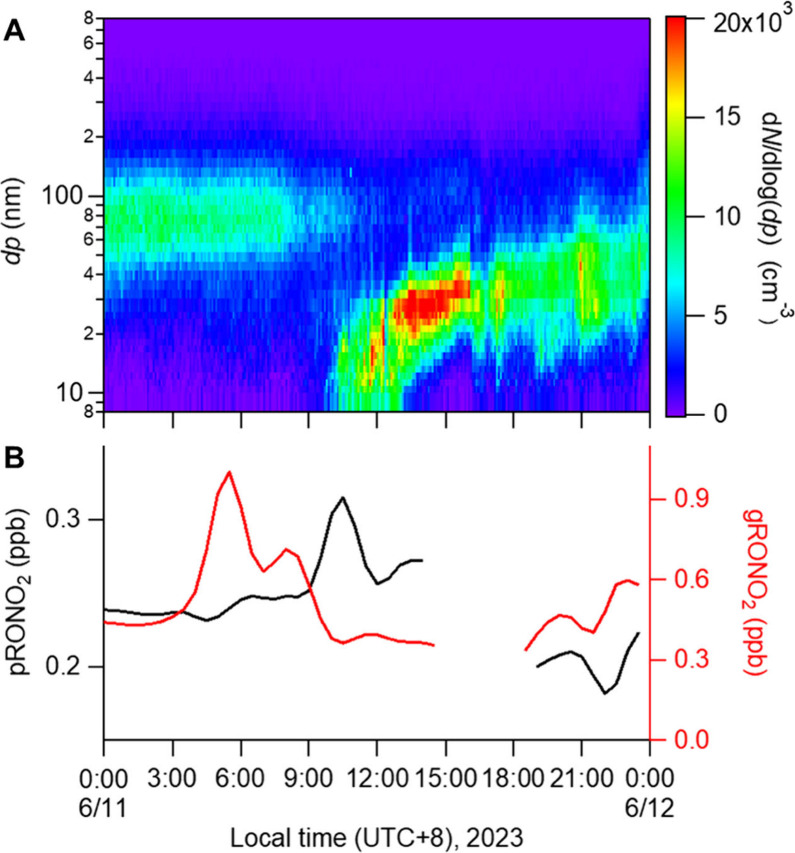
New particle formation (NPF) during Beijing summer 2023 campaign: (A) scanning mobility particle sizer (SMPS) measured size distributions with NPF events identified as surges in the concentrations of <10 nm particles and (B) alkyl nitrates (RONO_2_) in gas and particle phase measured by a thermal dissociation laser-induced fluorescence (TD-LIF).

#### ONs Formation in Stage II

Unlike stage I, stage II exhibited only particle growth without any occurrence of NPF events (Figure 2A). The SOA mass loading was approximately 300% greater than that in stage I. The chemistry in stage II was different from that in stage I. First, the daytime trend of NO_*z*_ concentration in stage II peaked at noon (Figure 1 and Figure S4), suggesting that photochemistry played a dominant role in NO_*z*_ formation unlike in stage I. Model simulations revealed that the most abundant RO_2_NO_2_ in stage II was PAN (up to 60% of RO_2_NO_2_), produced through the photooxidation of VOCs such as butane, methylglyoxal, and methyl vinyl ketone in the presence of NO_*x*_. PAN served as an important reservoir for reactive nitrogen species. Decomposition of PAN can significantly contribute to daytime O_3_ formation by releasing NO_2_ back into the atmosphere, particularly during warmer daytime conditions. We attributed the overestimation of RO_2_NO_2_, during the day, in both stage I (Figure 3) and II from the model (Figure S10) to missing lost mechanism for RO_2_NO_2_, due to the aerosol uptake and the gas-particle partitioning that are not included in F0AM–MCM.

## Atmospheric Implications

This study reveals that rapid nighttime production of ONs, dominated by gas-phase RONO_2_ from anthropogenic NO_*x*_, occurred in summertime urban air in Beijing. This process is critical for understanding the intricate dynamics of urban air pollution, as ONs played a central role in the formation of new particles and their subsequent growth, significantly influencing air quality. In addition, in stagnant air masses originating from the southeast North China Plain, we observed elevated concentrations of NO_*x*_ oxidation products (NO_*z*_) generated through NO_3_ radical-initiated oxidation of reactive terpenoids at the surface, or the residual layer followed by downward transport.

These findings have several important implications. They highlight that nocturnal ONs chemistry is a key driver of urban pollution episodes, as nighttime processes directly influence particulate composition and size distribution of the following day. The formation of ONs at night and their subsequent daytime transformation reflect the complex interplay between local anthropogenic emissions and regional transport. ONs are also related to the formation of tropospheric O_3_. Summer O_3_ pollution is one of the most challenging environmental issues in China, particularly since the implementation of the “Clean Air Act” in 2013.^41^ The chemistry behind the ONs formation (i.e., the reactions between RO_*x*_ and NO_*x*_) also produces O_3_. The production of ONs, therefore, suppresses O_3_ formation locally by providing a sink for HO_*x*_, RO_*x*_, and NO_*x*_, but the subsequent thermal decomposition and photochemistry of ONs increases O_3_ production.^42^ Current air quality models may underestimate SOA and O_3_ formation due to the omission of these nighttime pathways, highlighting the need to integrate nocturnal ONs production into predictive frameworks. The persistent underrepresentation of nocturnal organic nitrate chemistry in atmospheric models-particularly the NO_3_ initiated pathways that strongly modulate competition among oxidants-leads to systematic biases in simulated SOA yields and misrepresents the redistribution of NO_*x*_ that shapes next day ozone production.^2,11,43–45^ Moreover, with SO_2_ emissions declining globally as a result of effective control strategies, the relative role of nitrogen chemistry in shaping air quality is becoming increasingly important. This shift enhances the relevance of ONs formation and transformation in driving future urban air pollution, with direct implications for mitigation strategies targeting SOA and O_3_. Elevated nighttime NO_*x*_, especially from heavy-duty diesel traffic, enhances the formation of nitrate radicals and reactive nitrogen reservoirs that accelerate early morning SOA production and intensify ozone build-up after sunrise. These findings highlight nighttime NO_*x*_ control as a strategically powerful intervention point. Targeted mitigation of nocturnal emissions could substantially reduce daytime SOA and O_3_ peaks in urban environments undergoing a transition toward nitrogen-dominated oxidation regimes.

Another critical aspect is the role of vertical transport during the night, which efficiently redistributes ONs and their precursors. ONs generated in the residual layer can be preserved overnight and then entrained into the morning boundary layer, delivering reactive nitrogen and organic compounds to the surface. This downward mixing not only promotes early morning O_3_ and SOA production but also helps sustain pollution episodes under stagnant conditions. In parallel, the lofting of fresh anthropogenic emissions during the day enhances ONs production at higher altitudes, influencing regional aerosol burdens and long-range transport. ONs contribute to atmospheric brown carbon and may increase aerosol oxidative potential, raising concerns about adverse climate and health effects. These vertical exchange processes demonstrate how ONs integrate local surface processes with multiscale atmospheric chemistry, with implications extending from local air quality to regional climate and public health.

## Supplementary Material



## Data Availability

Data sets, including time series of species concentrations and meteorological variables during the campaign, are available at 10.5281/zenodo.10802900.

## Notes

Information about the modeling setting and figures to support the mechanism on organic nitrate formation at night (PDF)
