# Supplementary material for: Nighttime Organic Nitrates Drive New Particle Formation and Aerosol Growth in Urban Beijing
Source: Environ Sci Technol. 2026 Jul 7;60(28):19920–8. doi: 10.1021/acs.est.6c01200 (PMC13394426; doi:10.1021/acs.est.6c01200)
Supplement: Supplementary file 1 [file es6c01200_si_001.pdf]

Supplementary Materials of

Nighttime Organic Nitrates Drive New Particle Formation and Aerosol Growth in Urban Beijing

Junfeng Wang[1,2], Eleonora Aruffo[3,*], Jianhuai Ye[2,4,*], Xinlei Ge[5,*], Scot T. Martin[2], Yele Sun[6], Weiqi Xu[6], Yuepeng Pan[6], Alessandra Mascitelli[7], Piero Chiacchiaretta[7], Jie Zhang[8], Jingyi Li[1], Yiming Qin[9], Haiwei Li[1], Ke Li[1], Ning Zhang[1], Ming Wang[1], Chunxiang Ye[10], Jianbo Zhang[10], Wei Tao[4], Tzung-May Fu[4], Hong Liao[1], Pingqing Fu[11], Qi Zhang[12], Mindong Chen[1], Jingkun Jiang[13], Hugh Coe[14], David Topping[14], James Lee[15], Piero Di Carlo[7], and Daniel J. Jacob[2]

[1]Collaborative Innovation Center of Atmospheric Environment and Equipment Technology, Joint International Research Laboratory of Climate and Environment Change (ILCEC), School of Environmental Science and Engineering; Nanjing University of Information Science and Technology; Nanjing, 210044, China.

[2]John A. Paulson School of Engineering and Applied Sciences, Harvard University; Cambridge, MA 02138, USA.

[3]Department of Science, University "G. d'Annunzio" of Chieti-Pescara, Center for Advanced Studies and Technology-CAST; Chieti, 66100, Italy.

[4]Guangdong Provincial Observation and Research Station for Coastal Atmosphere and Climate of the Greater Bay Area (GORSCAC), School of Environmental Science & Engineering, Southern University of Science and Technology; Shenzhen, 518055, China.

[5]School of Energy and Environment, Southeast University Nanjing, 211189, China

[6]State Key Laboratory of Atmospheric Boundary Layer Physics and Atmospheric Chemistry, Institute of Atmospheric Physics, Chinese Academy of Sciences; Beijing, 100029, China.

[7]Department of Advanced Technologies in Medicine & Dentistry, University "G. d'Annunzio" of Chieti-Pescara, Center for Advanced Studies and Technology-CAST; Chieti, 66100, Italy.

[8]Atmospheric Sciences Research Center, University at Albany, State University of New York; Albany, NY 12203, USA.

[9]School of Energy and Environment, City University of Hong Kong; Hong Kong 999077, China.

[10]State Key Joint Laboratory of Environmental Simulation and Pollution Control, College of Environmental Sciences and Engineering, Peking University; Beijing, 100080, China.

[11]Institute of Surface-Earth System Science, Tianjin University; Tianjin, 300072, China

[12]Department of Environmental Toxicology, University of California Davis; Davis, CA 95616, USA.

[13]School of Environment, Tsinghua University; Beijing, 100084, China.

[14]School of Earth and Environmental Science, University of Manchester; Manchester M13 9PL, UK

[15]Department of Chemistry, University of York, York YO10 5DD, UK

*Corresponding authors:
eleonora.aruffo@unich.it; yejh@sustech.edu.cn; caxinra@163.com

Total of page: 14; number of figures: 10; number of tables: 2

Text S1 WRF-Chem simulation of NO_3 flux

The WRF-Chem (Weather Research and Forecasting model coupled with Chemistry, Version 3.8) simulations were conducted from 00:00 local time (UTC+8) on July 2 to 23:00 on July 30, 2019, covering a 29-day summer period to analyze atmospheric chemical processes during typical warm-season conditions. The model domain spanned a broad East Asian region from 92.70°E to 133.30°E longitude and from 17.70°N to 48.23°N latitude, employing a horizontal resolution of 30 km × 30 km sufficient for regional-scale analysis while maintaining computational efficiency, with 30 vertically stratified layers that provided enhanced resolution in the lower atmosphere to better capture near-surface processes. Model outputs were specifically analyzed for a focal urban location (116.47°E, 39.91°N, corresponding to the Beijing metropolitan area), where the vertical flux of NO_3 radical, which is a key oxidant that dominate nighttime atmospheric chemistry, was quantitatively evaluated between the surface layer (0-31 m) representing immediate ground-level interactions and merged layers 2-4 (31-233 m) encompassing the typical nocturnal boundary layer height in this region. The monthly average diurnal variation was plotted to characterize temporal patterns. Positive flux values indicate net upward transport (surface-to-atmosphere) of these oxidants, likely resulting from near-ground chemical production, while observed negative values would suggest downward transport processes including turbulent diffusion, dry deposition to surfaces, or chemical depletion under stable atmospheric conditions typical of nighttime periods. This modeling framework incorporated meteorological reanalysis data for dynamical constraints and the RACM (Regional Atmospheric Chemistry Mechanism) chemical mechanism to accurately represent nighttime boundary layer chemistry and transport processes, providing valuable insights into the vertical exchange of reactive species and the nocturnal oxidation capacity that significantly influences nighttime atmospheric chemistry.

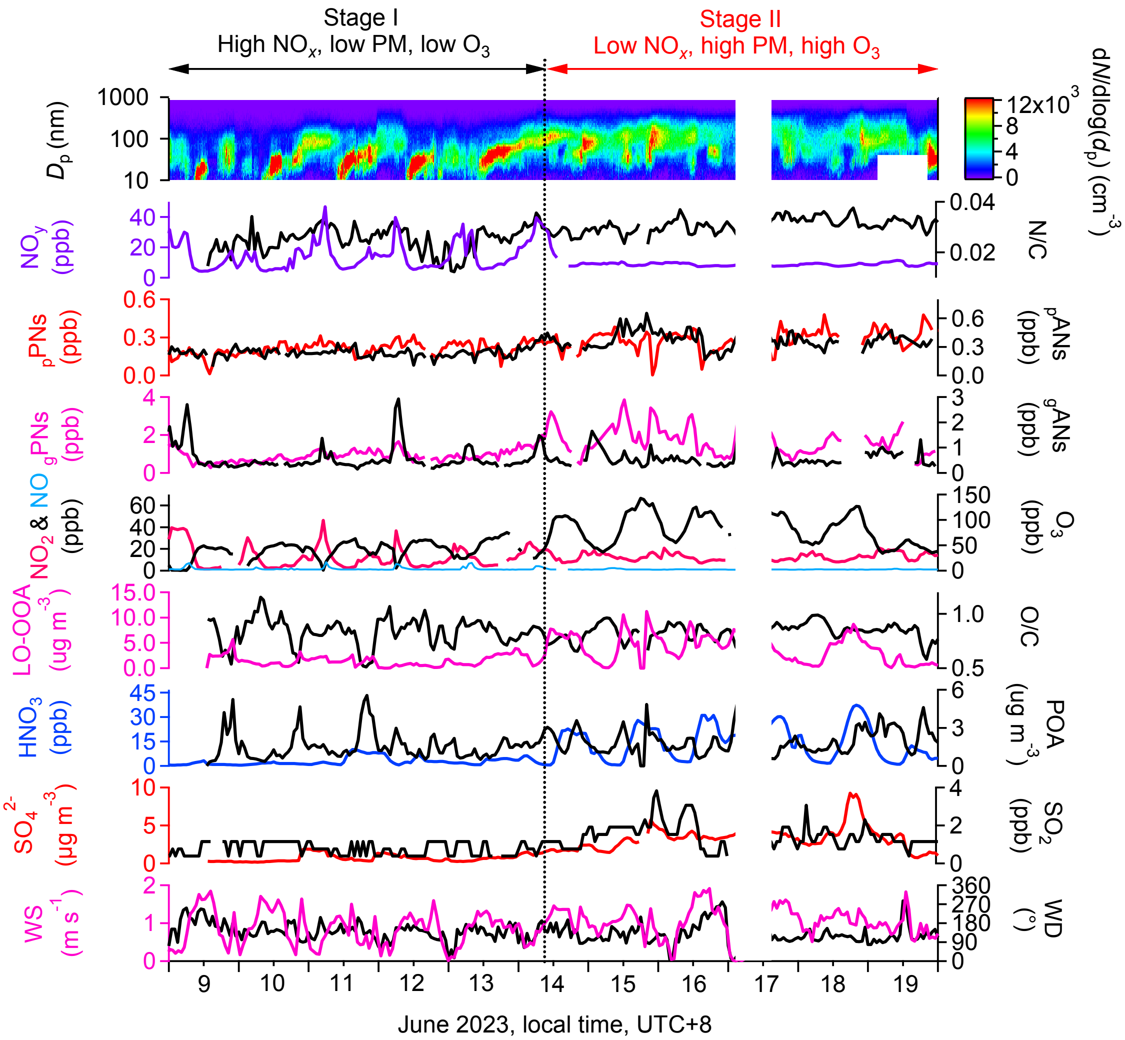

Fig. S1 Meteorological conditions and atmospheric composition measurements during June 2023 at the IAP tower, i.e. same site of the 2017 summer campaign in Beijing. NO_2, total peroxyl nitrate both in gas and particle phase (gPNs and pPNs) and total alkyl nitrate both in gas and particle phase (gANs and pANs) have been measured by a TD-LIF instrument. HNO_3 is retrieved from the Goddard Earth Observing System Composition Forecast (GEOS-CF) system, which is a high-resolution (0.25°) global constituent prediction system from NASA's Global Modeling and Assimilation Office (GMAO) (*1*).

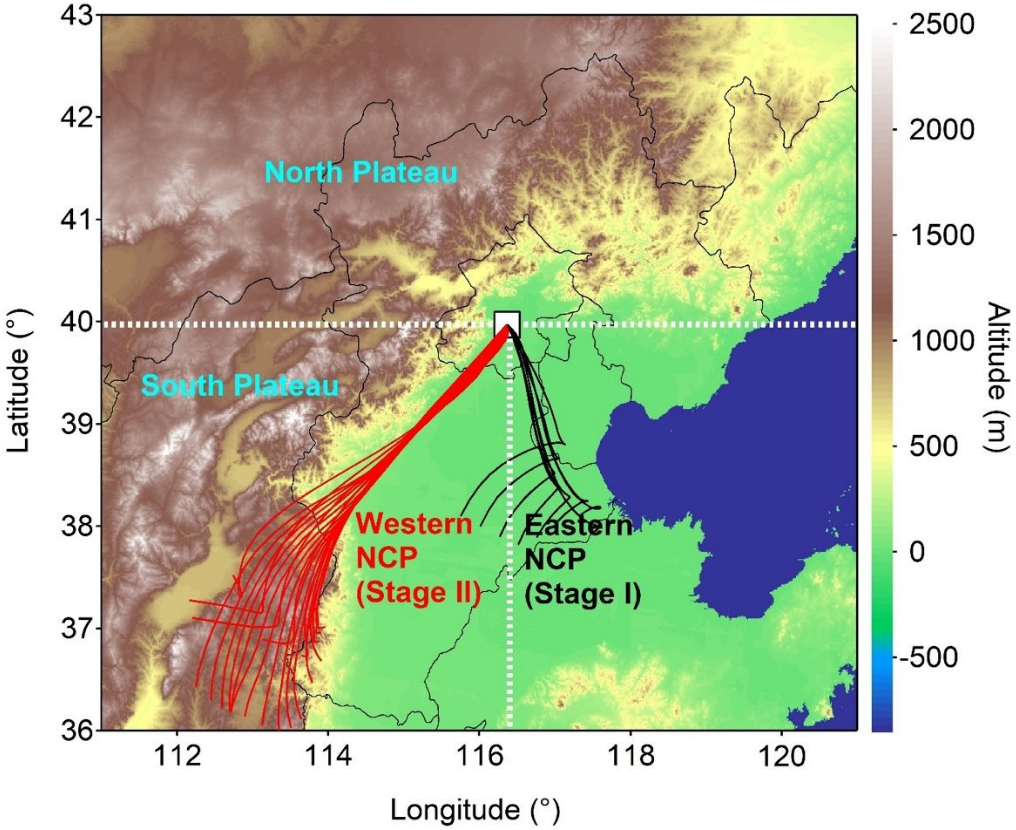

Fig. S2 Sampling site (white solid square) and the regional classification based on the 24h back trajectories analysis and the emission characteristics.

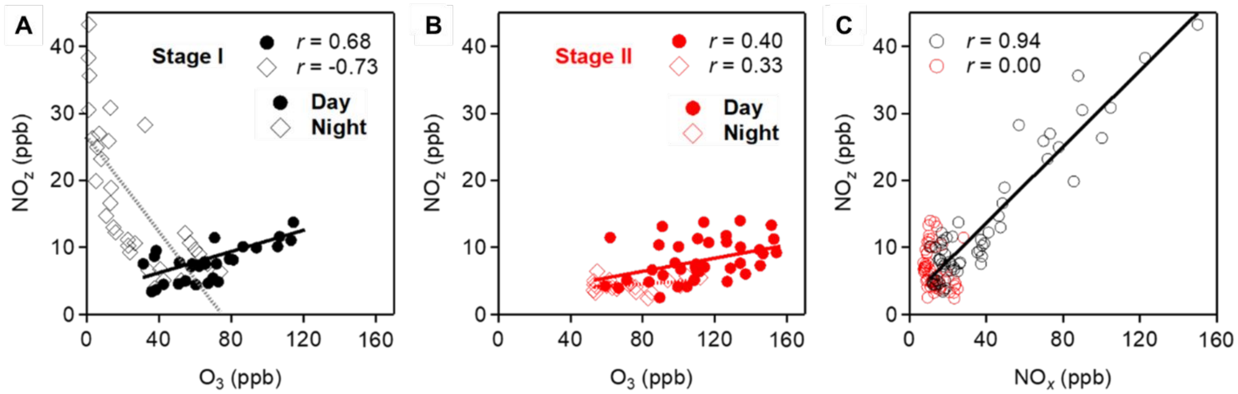

Fig. S3 Scatter plots of NO_z versus (a-b) O_3, and (c) NO_x in two different stages. Data are shown for Stage I (black) and II (red) of June 9-20, 2017, and separately for day and night. Individual points are hourly mean values.

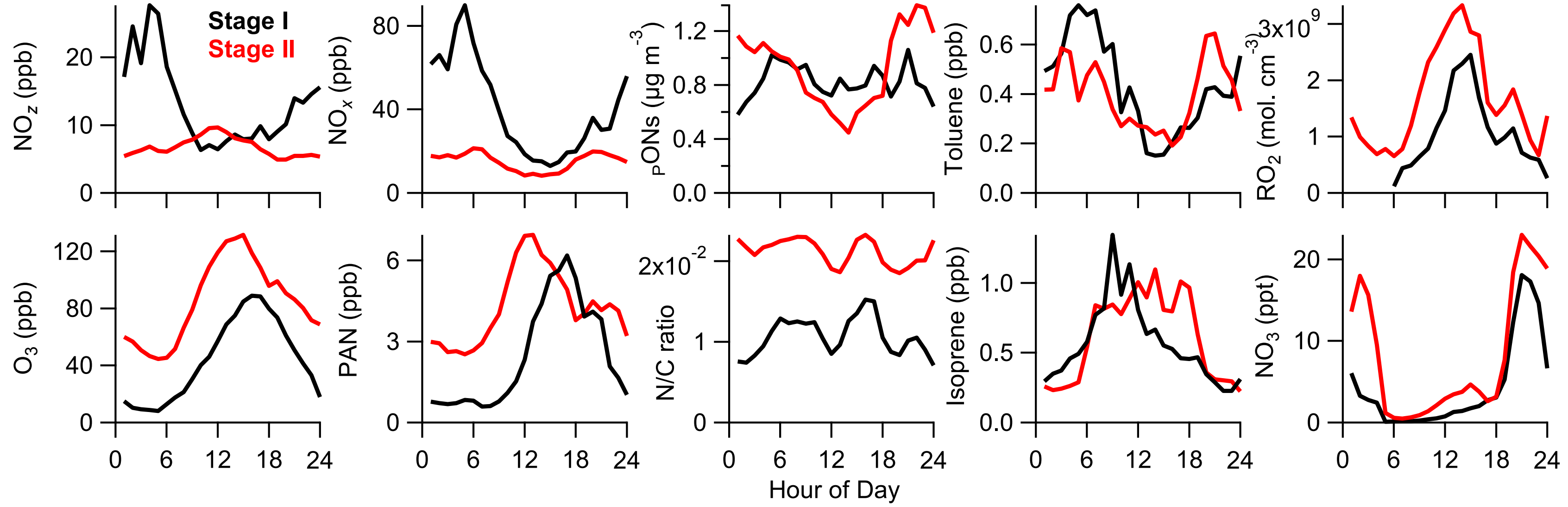

Fig. S4 Diurnal mean during Stage I and Stage II for the most significant species in analysis.

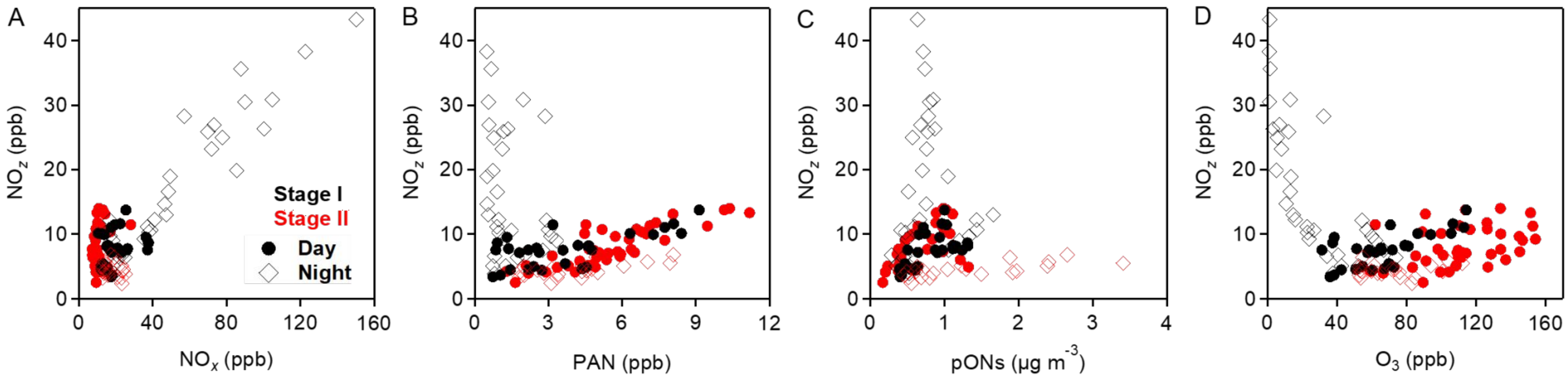

Fig. S5 Scatterplots of the concentrations of NO_x oxidation products (NO_z) versus (A) NO_x; (B) PAN; (C) pON_s; (D) O_3. Data are shown for Stage I (black) and II (red) of June 9-20, 2017, and separately for day and night. Individual points are hourly mean values.

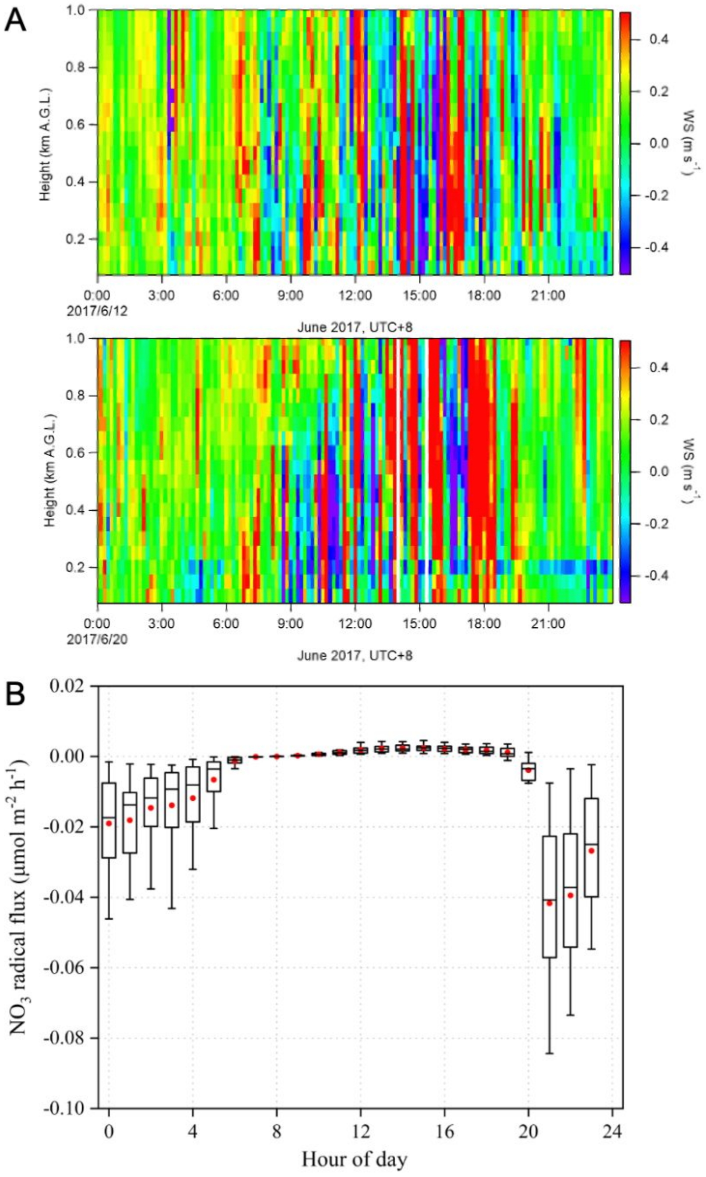

Fig. S6 (A) Vertical wind speed profiles on June 12, 2017 (Stage I) and June 20, 2017 (Stage II). (B) Monthly average diurnal variation of NO_3 flux between the surface layer and the near surface atmosphere during summer Beijing 2019. Positive flux values indicate net upward transport (surface-to-atmosphere), while observed negative values suggest downward transport processes.

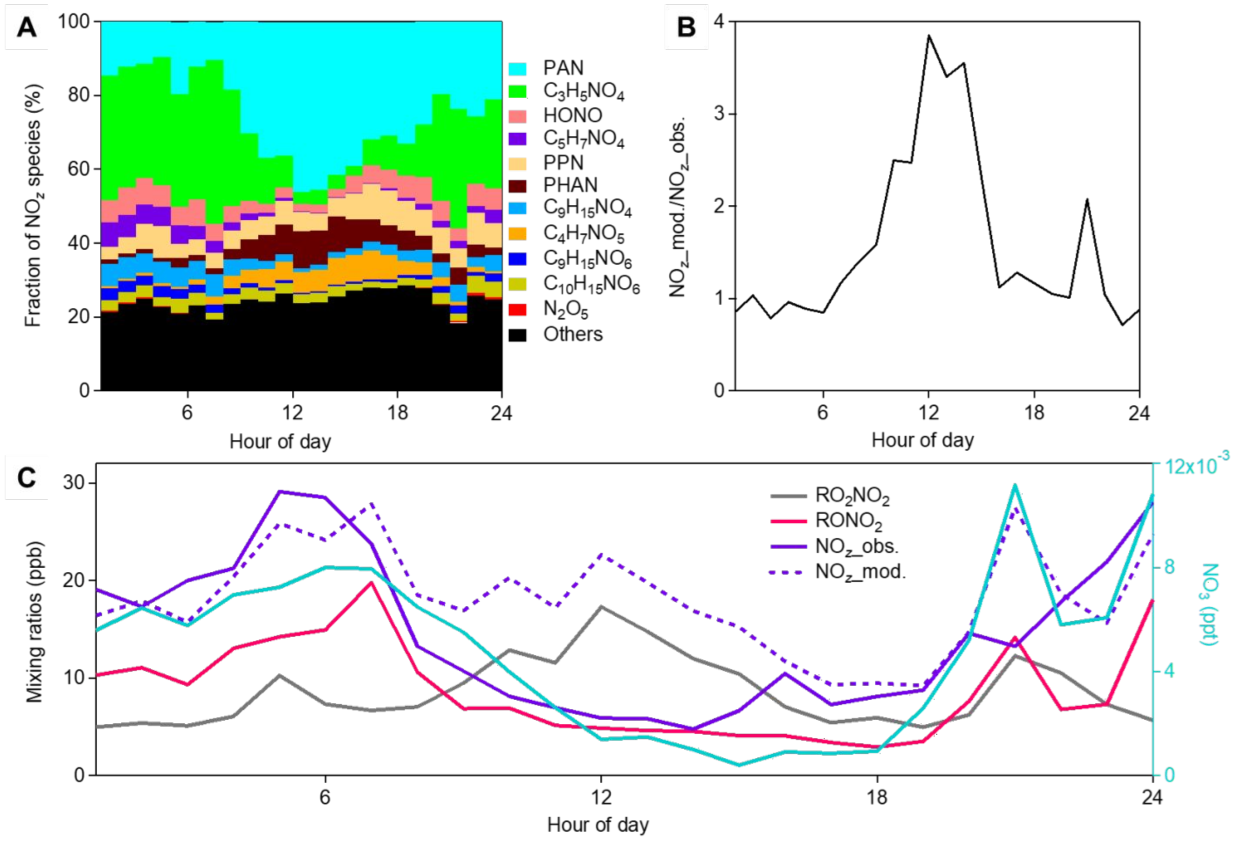

Fig. S7 MCM model simulations for Stage I. (A) Speciation of the ONs from MCM simulation that includes isoprene as input; (B) ratio of modeled to measured total NO_z; (C) the measured NO_z and simulated NO_z.

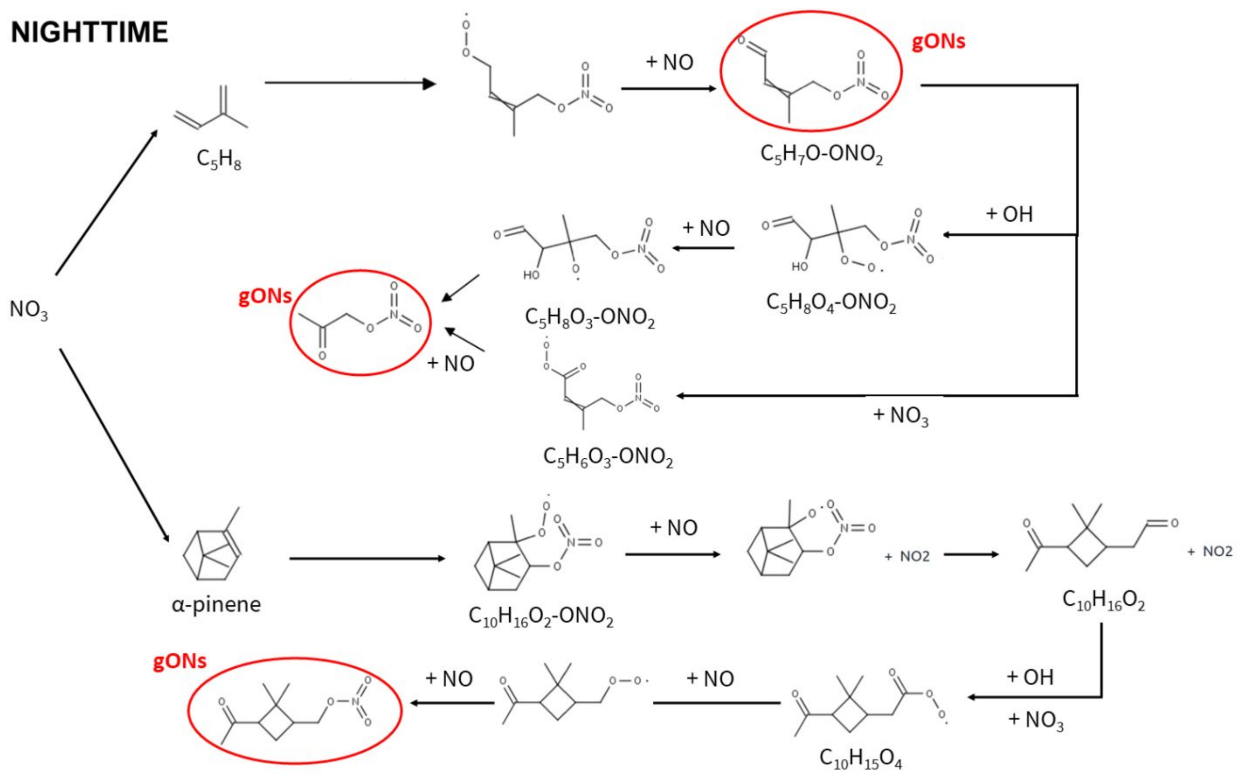

Fig. S8 The proposed mechanism for the nocturnal production of gas phase ONs. The mechanism has been developed on the basis of the F0AM 0D box model based on the MCM mechanism to evaluate the gas phases chemistry in Beijing in Stage I. These results obtained from this simulation has been used to constrain the F0AM model with the integration of the WAM module, to simulate also the particle phase, focusing on this case in the gas-to-particle partitioning of ONs.

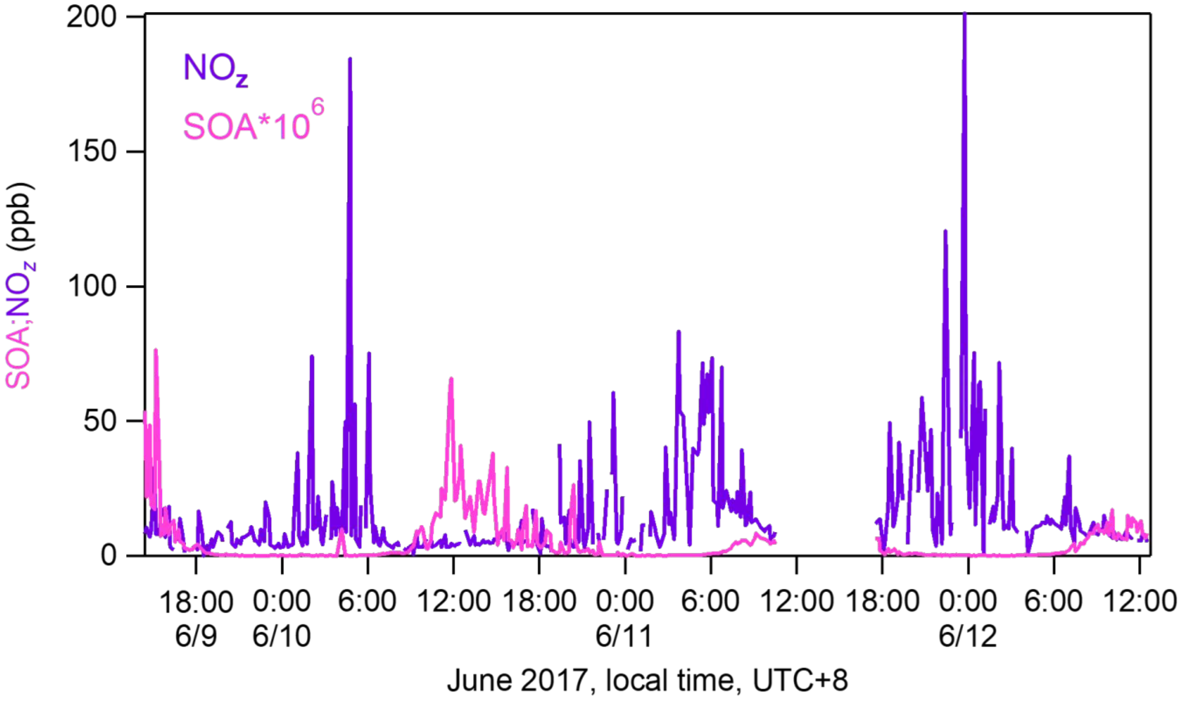

Fig. S9 F0AM-WAM simulation results in Stage I. Particles concentration (pink line) peaked during the daytime, as the nocturnal NO_z (purple line) is photolyzed after the sunrise.

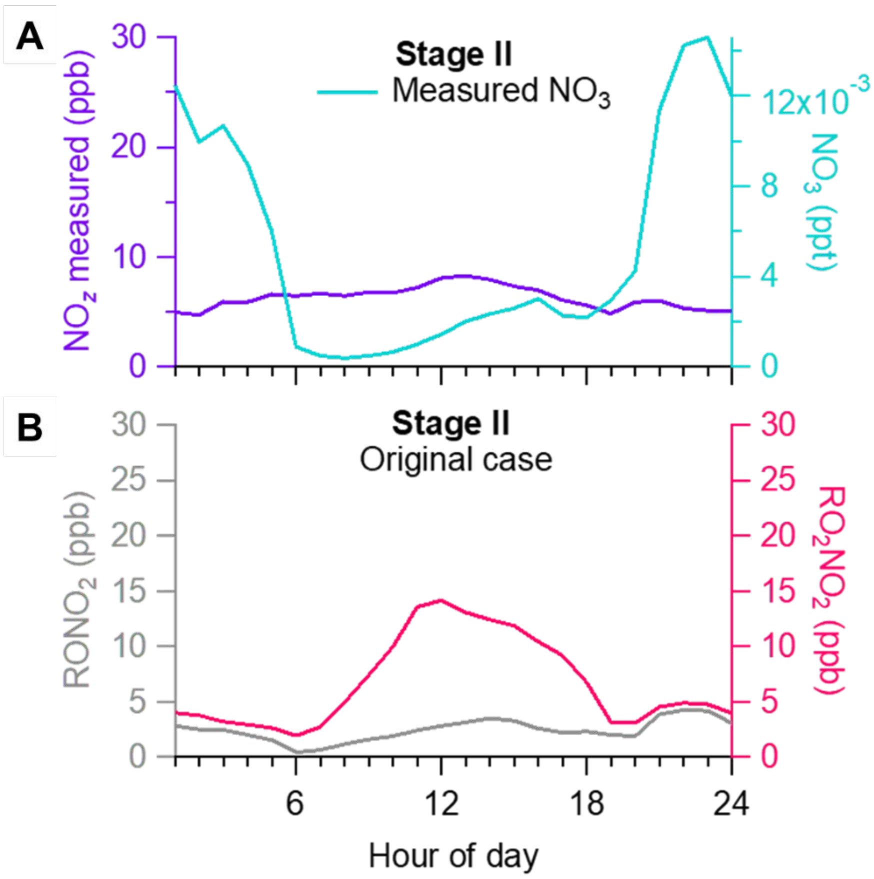

Fig. S10 MCM model simulations for Stage II. (A), the measured NO_z and NO_3; (B), the $RONO_2$ and RO_2NO_2 simulated with the measured NO_3 (the original case).

Table S1 Summary for the measured compounds significant for the present analysis with mean and standard deviation in summer 2017.

	Day		Night	
Variables	Stage I	Stage II	Stage I	Stage II
NO_z (ppb)	9.5 ± 5.5	7.6 ± 3.2	17.6 ± 10.7	5.7 ± 1.9
NO (ppb)	7.7 ± 14.4	0.7 ± 0.6	14.5 ± 19.5	0.2 ± 0.16
NO_2 (ppb)	21.8 ± 10.9	12.5 ± 5.7	38.6 ± 18.4	17.5 ± 4.0
pONs (ppb)	0.30 ± 0.13	0.27 ± 0.17	0.29 ± 0.13	0.44 ± 0.23
HONO (ppb)	0.63 ± 0.33	0.72 ± 0.30	1.15 ± 0.54	1.06 ± 0.35
N_2O_5 (ppt)	10.8 ± 17.2	7.2 ± 7.9	121.4 ± 143.2	98.7 ± 58.6
NO_3 (ppt)	1.5 ± 1.9	2.7 ± 2.4	9.2 ± 12.0	16.2 ± 11.1
$ClNO_2$ (ppt)	-	-	-	-
HNO_3 (ppb)	-	-	-	-
PAN (ppb)	3.1 ± 2.5	4.9 ± 2.3	1.8 ± 2.0	3.4 ± 1.6
O_3 (ppb)	56.5 ± 29.8	98.4 ± 32.2	28.1 ± 23.0	67.4 ± 19.1
N/C	0.012 ± 0.004	0.021 ± 0.003	0.009 ± 0.003	0.021 ± 0.002
OSc	-0.67 ± 0.30	-0.21 ± 0.14	-0.78 ± 0.27	-0.25 ± 0.13
OA $Size_{Peak}$ (nm)	475 ± 124	559 ± 44	463 ± 106	547 ± 59
SOA (μg m^{-3})	3.77 ± 1.87	10.38 ± 4.47	4.39 ± 2.40	13.48 ± 7.93
*r*BC (μg m^{-3})	1.80 ± 1.39	1.55 ± 0.81	2.25 ± 1.28	1.89 ± 0.70
$Sum\_{PAN+pONs+HONO+NO3}$ (ppb)	4.03 ± 2.96	5.89 ± 2.77	3.24 ± 2.67	4.9 ± 1.18

Table S2 Summary for the measured compounds used as input to constrain the F0AM model.

**Input species for the F0AM model (ppb)**
O_3
CO
OH
NO
NO_2
NO_3
N_2O_5
HONO
Isoprene
Benzene
Toluene
Acetone
Acetaldehyde
Methanol
n-Butane
Ethanol
n-Heptane
1,3-Butadiene
n-Pentane
1-Butene
1-Pentene
Acetylene
Propane
Ethene
Ethane
α-pinene*
M-xylene
P-xylene
HNO_3**

*α-pinene has been approximated as a fraction of C_5H_8, due to its good correlation with C_5H_8 found in 2023.
**HNO_3 has not been measured. Sensitivity tests varying the HNO_3 have been done, founding that HNO_3 does not impact on the manuscript results.

References

1. Keller, C. A., Knowland, K. E., Duncan, B. N., Liu, J., Anderson, D. C., Das, S., ... & Pawson, S. (2021). Description of the NASA GEOS composition forecast modeling system GEOS-CF v1. 0. Journal of Advances in Modeling Earth Systems, 13(4), e2020MS002413. doi:10.1029/2020MS002413
